# Prediction of prognosis, immune infiltration and immunotherapy response with N6-methyladenosine-related lncRNA clustering patterns in cervical cancer

**DOI:** 10.1038/s41598-022-20162-2

**Published:** 2022-10-14

**Authors:** Haixia Jia, Meiting Cao, Suhua Hao, Jiahao Wang, Jintao Wang

**Affiliations:** 1 Department of Scientific Research, Shanxi Province Cancer Hospital, Taiyuan, Shanxi China; 2 Department of Gynecology, Shanxi Province Cancer Hospital, Taiyuan, Shanxi China; 3 Department of Prevention Care, Shanxi Province Cancer Hospital, Taiyuan, Shanxi China; 4 Department of Epidemiology, School of Public Health, Shanxi Medical University, 56, Xinjian Nan Road, Taiyuan, China

**Keywords:** Cancer, Genetics, Immunology, Biomarkers, Oncology

## Abstract

LncRNAs and tumor microenvironment (TME) exert an important effect in antitumor immunity. Nonetheless, the role of m^6^A-related lncRNA clustering patterns in prognosis, TME and immunotherapy of cervical cancer (CC) remains unknown. Here, based on 7 m^6^A-related prognostic lncRNAs obtained from TCGA-CC dataset, two m^6^AlncRNA clustering patterns were determined. m^6^AlncRNA clusterA was characterized by immune cell infiltrates and immune activation. m^6^AlncRNA clusterB was characterized by enrichment of immune evasion and tumorigenic activation pathways as well as survival and clinical stage disadvantage. Then, principal component analysis algorithms were used to construct m^6^AlncRNAscore based on prognostic differentially expressed genes between two m^6^AlncRNA clusters to quantify m^6^AlncRNA clustering patterns. m^6^AlncRNAscore was an independent prognostic protective factor. Higher Th2 and Treg cells and enrichment of immunosuppressive pathways were observed in the low-m^6^AlncRNAscore group, with poorer survival. High-m^6^AlncRNAscore was characterized by increased infiltration of activated CD8 T cell, enrichment of immune activation pathways, lower IL-10 and TGF-beta1 levels, and higher immunophenscore values, indicating inflamed TME and better anti-tumor immunotherapy efficacy. Quantitative Real-Time Polymerase Chain Reaction was used for detection of m^6^A-related prognostic lncRNAs. Collectively, we identified two m^6^AlncRNA clustering patterns which play a nonnegligible role in the prognosis, TME heterogeneity and immunotherapy of CC patients.

## Introduction

Cervical cancer (CC) is the fourth leading cause of cancer deaths in women globally^1^. In China, 59,000 women died of CC in 2020, accounting for about 17% of all CC deaths worldwide in the same year^2^. High mortality of CC is associated with recurrence and an advanced stage at diagnosis. Patients with recurrent and/or advanced CC have limited treatment options and poor prognosis, with a 5-year survival probability of 17%^3^. Immunotherapy represented by immune checkpoint inhibitors (ICIs) provides a promising perspective for cancer treatment. However, the overall response rate of ICIs was only 10–25% in previous clinical trials for CC^4^. The mechanisms behind the poor response of ICIs deserve further investigation. Increasing evidence indicates that tumor microenvironment (TME) not only influences tumor cell growth and metastasis, but also strongly affects tumor immune escape and immunotherapy efficacy^5,6^. Therefore, the heterogeneity and complexity of TME and novel biomarkers associated with TME should be further explored to predict immunotherapeutic response and provide new therapeutic targets for tumors.

N6-methyladenosine (m^6^A) modification, the most common epigenetic modification in eukaryotic messenger RNAs and long non-coding RNAs (lncRNAs), plays a crucial role in RNA processing, splicing, degradation, and translation, thereby affecting cell self-renewal, differentiation, tumorigenesis, and tumor progression^7–9^. m6A modification is a dynamic reversible process regulated by methyltransferases, binding proteins, and demethylases^10^. Methyltransferases are composed of METTL3/14/16, ZC3H13, RBM15, WTAP, VIRMA, and RBM15B, and catalyze the RNA methylation modification process^10–12^. Demethylases include FTO^12^ and ALKBH3/5^12^, and mediate the RNA methylation removal process. Binding proteins consist of YTHDF1/2/3, YTHDC1/2, HNRNPA2B1, LRPPRC, FMR1, TRMT112, ZCCHC4, NUDT21, CPSF6, SETD2, SRSF3, SRSF10, XRN1, NXF1, PRRC2A, IGF2BP1/2/3, IGFBP3, and RBMX, exerting a vital role in carcinogenesis, invasion, and metastasis by combining with m^6^A motif^10–12^.

Although over 85% of the human genome is transcribed, less than 3% of the transcripts encode protein, and the remaining transcripts mainly are non-coding RNAs^13^. LncRNAs, more than 200 nucleotides in length, constitute the largest group of ncRNAs and play a key role in transcriptional and post-transcriptional regulation^14^. It has been reported that lncRNAs exert an important effect on oncogenesis, metastasis, TME, and tumor immune escape and might be potential therapeutic targets for cancer^14–18^. However, the relationship between m^6^A-related lncRNA clustering patterns and TME immune infiltration remains unclear.

In this study, we established a scoring system, m^6^AlncRNAscore, to quantify the m^6^AlncRNA clustering patterns in individual patients with CC. Further, we explored the independent prognostic value of m^6^AlncRNAscore in the overall survival (OS), progression-free survival (PFS), and disease-specific survival (DSS), and the potential predictive role in immunotherapy efficacy. Additionally, we explored the correlation of m^6^AlncRNA clustering patterns with clinicopathologic characteristics, TME immune infiltration, and somatic mutation, as well as the potential mechanisms in CC. Finally, we validated the expression of 4 m^6^A-related prognostic lncRNAs in tumor samples and normal tissues.

## Materials and methods

### Data acquisition

The RNA sequencing and somatic mutation data were downloaded from the Cancer Genome Atlas (TCGA, https://portal.gdc.cancer.gov/) database. The immunophenscore (IPS) data were downloaded from the Cancer Immunome Atlas (TCIA, https://tcia.at/) database. The clinical information was downloaded from UCSC Xena (https://xenabrowser.net/). The sequencing data of 306 CC samples and 3 normal tissues were downloaded. The clinical information was summarized in Supplementary Table 1. Patients with OS less than or equal to 30 days were excluded, leaving the remaining 273 CC patients were enrolled into the further survival analysis. All methods were performed in accordance with the relevant guidelines and regulations.

### Identification of m^6^A-related lncRNAs

The transcriptome sequencing genes were divided into mRNA genes and lncRNA genes according to the human genome annotation data. Expression levels of 34 m^6^A regulators were extracted from the mRNA data. Pearson correlation coefficient was then used to assess the correlation between m^6^A regulators and lncRNAs. The lncRNAs with absolute correlation coefficient > 0.40 and *P* value < 0.001 were regarded as m^6^A-related lncRNAs. Next, univariate Cox regression analysis was applied to determine lncRNAs associated with prognosis. The m^6^A-related lncRNAs with *P* value < 0.05 were considered as m^6^A-related prognostic lncRNAs.

### Consensus clustering analysis

The “ConsensusClusterPlus” package (1000 iterations) was utilized to divide patients into different clustering patterns, referred to as m^6^AlncRNA clusters, based on the expression levels of m^6^A-related prognostic lncRNAs. According to the expression of prognosis-associated DEGs between different m^6^AlncRNA clusters, patients were again classified into different clustering subtypes, termed as gene clusters.

### Gene set variation analysis (GSVA)

To explore the difference of biological process activity between different subgroups, we conducted GSVA enrichment analysis by using the “GSVA” package. The “c2.cp.kegg.v7.4.symbols” gene sets were downloaded from MSigDB database for running GSVA analysis. Adjusted *P* value < 0.05 was regarded as statistically significant.

### Estimation of TME immune cell infiltration

The single-sample gene-set enrichment analysis (ssGSEA) algorithm was used to quantify the relative infiltration levels of TME immune cells. The gene set for marking 23 immune cell types was acquired from the published study^19,20^. The ssGSEA score was applied to represent the relative abundance of each infiltrating immune cell in each patient. Moreover, ESTIMATE algorithm was utilized to quantify the stromal and immune components for each patient.

### Identification of DEGs and KEGG pathway enrichment analysis

The “limma” package was utilized to determine differentially expressed genes (DEGs) between different m^6^AlncRNA clusters. The significance criterion for determining DEGs was set as adjusted *P* value < 0.001. The “clusterProfiler” package was employed to perform KEGG enrichment analysis for the DEGs to explore their potential biological behaviors.

### Generation of m^6^AlncRNAscore

To further investigate the role of m^6^AlncRNA clustering patterns in CC, we constructed a scoring system, namely m^6^AlncRNAscore, for individual patients based on the prognosis-associated DEGs between different m^6^AlncRNA clusters. The procedures for m^6^AlncRNAscore establishment were as follows: first, we extracted prognosis-associated DEGs by univariate Cox regression analysis; second, principal component analysis (PCA) was used to construct m^6^AlncRNAscore based on each prognostic DEG expression for each sample. The advantage of this method is that the score is focused on the largest well-correlated (or anti-correlated) gene block in the set, while the contribution weight from genes that are not tracked with other set is reduced. Similar to GGI establishment^20,21^, the m^6^AlncRNAscore formula was as follows:

$$ m^{6} AlncRNAscore = \mathop \sum \limits_{1}^{n} (PC1_{i} + PC2_{i } ) $$

where n is the total number of prognosis-associated DEGs, and i is the expression of the ith prognostic DEG.

### Somatic mutation analysis

The “maftools” package was used to analyze the somatic mutation data of patients. Tumor mutation burden (TMB), mutations per million bases, was calculated for each patient. Then, we compared TMB between different m^6^AlncRNAscore groups.

### Prediction of response to ICIs

TCIA database provides immune profiles and antigenomes for 20 solid tumors including CC. IPS ranges from 0 to 10, represents tumor immunogenicity. The larger the IPS value, the stronger the immunogenicity. It has been validated that IPS could predict the response of tumor patients to ICIs^19–23^.

### Drug sensitivity prediction

We predicted the chemotherapeutic drug sensitivity based on the Genomics of Drug Sensitivity in Cancer (GDSC) database (https://www.cancerrxgene.org/). oncoPredict package was used to estimate the half-maximal inhibitory concentration (IC50).

### Construction of ceRNA network

We firstly obtained the miRNAs interacting with the m^6^A-related prognostic lncRNAs by co-expression method. The miRNAs with absolute correlation coefficient < -0.20 and *P* value < 0.001 were regarded as related miRNAs. Then, we predicted the miRNA target genes (mRNA) by miRanda, miRDB, miRTarBase and TargetScan software. When all four kinds of software consider this gene as the target gene of miRNA, we regard this gene as the final target gene. The lncRNA-miRNA and miRNA-mRNA regulatory relationships were integrated to construct the competing endogenous RNA (ceRNA) network using Cytoscape software.

### Sample collection

We totally collected 14 cervical tissue specimens, including 6 cervical cancer samples and 6 healthy controls in the Gynecology Department of Cancer Hospital Affiliated to Shanxi Medical University. Ethical approval was obtained from the Science Research Ethics Committee of Cancer Hospital Affiliated to Shanxi Medical University (No: SJJ202105). Informed consent and approval were provided by all participants. 6 patients with cervical cancer were newly diagnosed FIGO stage I/II patients without receiving any treatment.

### Quantitative real-time polymerase chain reaction (qRT-PCR)

Total RNA was isolated from 12 samples using RNA TRIzol reagent (Tiangen Biotech Co., Ltd., Beijing, China, #DP451). cDNA synthesis was conducted with PrimeScriptTM RT Master Mix (Takara Biomedical Technology Co., Ltd., Beijing, China, #RR036Q). Real-time PCR was then performed with TB Green Premix Ex Taq (Takara Biomedical Technology Co., Ltd., Beijing, China, #RR820A). Relative expression of lncRNAs were normalized to GAPDH and calculated by 2-ΔΔCt method. Primers sequences are listed in Supplementary Table 2.

### Statistical analysis

All statistical analyses were done in R version 4.0.4. Pearson correlation test was employed for assessing the relationship between m^6^A regulators and lncRNAs. Wilcoxon rank sum test was applied to compare the quantitative data such as m^6^A-related prognostic lncRNAs, immune cell infiltration, and m^6^AlncRNAscore between groups. Kaplan–Meier method was utilized to draw survival curves, and log-rank test was performed to compare the survival difference between groups. The predictive accuracy of m^6^AlncRNAscore was evaluated using the receiver operating characteristic (ROC) curve and area under curve (AUC). Multivariable Cox regression model was applied to ascertain the independent prognostic factors of CC. According to the association between m^6^AlncRNAscore, TMB, and OS, we used the “survminer” package to find the optimal cutoff values of m^6^AlncRNAscore and TMB, respectively. Patients were then divided into different groups according to the optimal cutoff value. Unless otherwise specified, a two-sided *P* value < 0.05 was considered statistically significant.

## Results

### Identification of m^6^A-related lncRNAs

Pearson correlation analysis was used to assess the relationship between 14,086 lncRNAs and 34 m^6^A regulators. Total 112 lncRNAs with absolute correlation coefficient > 0.40 and *P* value < 0.001 were considered as m^6^A-related lncRNAs. Univariate Cox regression analysis was used to explore the prognostic roles of m^6^A-related lncRNAs. Of the 112 m^6^A-related lncRNAs, 7 were associated with the OS (Table 1). These results indicated that the 7 m^6^A-related lncRNAs, including AC024270.4, AC008124.1, AL109811.2, AC015922.2, AC099850.4, AC025176.1, and RPP38-DT, might be potential prognostic biomarkers of CC, termed as m^6^A-related prognostic lncRNAs.

**Table 1:** m^6^A-related lncRNAs associated with prognosis of cervical cancer.

m^6^A-related prognostic lncRNAs	HR (95% CI)	*P* value
AC024270.4	0.048 (0.004, 0.581)	0.017
AC099850.4	1.042 (1.007, 1.079)	0.018
AC025176.1	0.806 (0.659, 0.984)	0.034
AC008124.1	0.628 (0.422, 0.935)	0.022
AL109811.2	0.801 (0.672, 0.954)	0.013
AC015922.2	1.088 (1.022, 1.159)	0.009
RPP38-DT	0.068 (0.005, 0.838)	0.036

### Expression profiles of m^6^A-related prognostic lncRNAs

To explore the potential biological function of m^6^A-related lncRNAs in the occurrence of CC, we compared the expression profiles of 7 m^6^A-related prognostic lncRNAs between CC samples and normal tissues. Notably, the tumor samples showed significantly lower expression levels of AC024270.4, AC008124.1, AL109811.2, and AC015922.2, but higher levels of AC099850.4, AC025176.1, and RPP38-DT, compared with the normal samples (Supplementary Fig. 1). These findings suggested that the 7 m^6^A-related prognostic lncRNAs might possess important biological roles in the development of CC.

### Consensus clustering patterns of m^6^A-related prognostic lncRNAs

**Figure 1:**
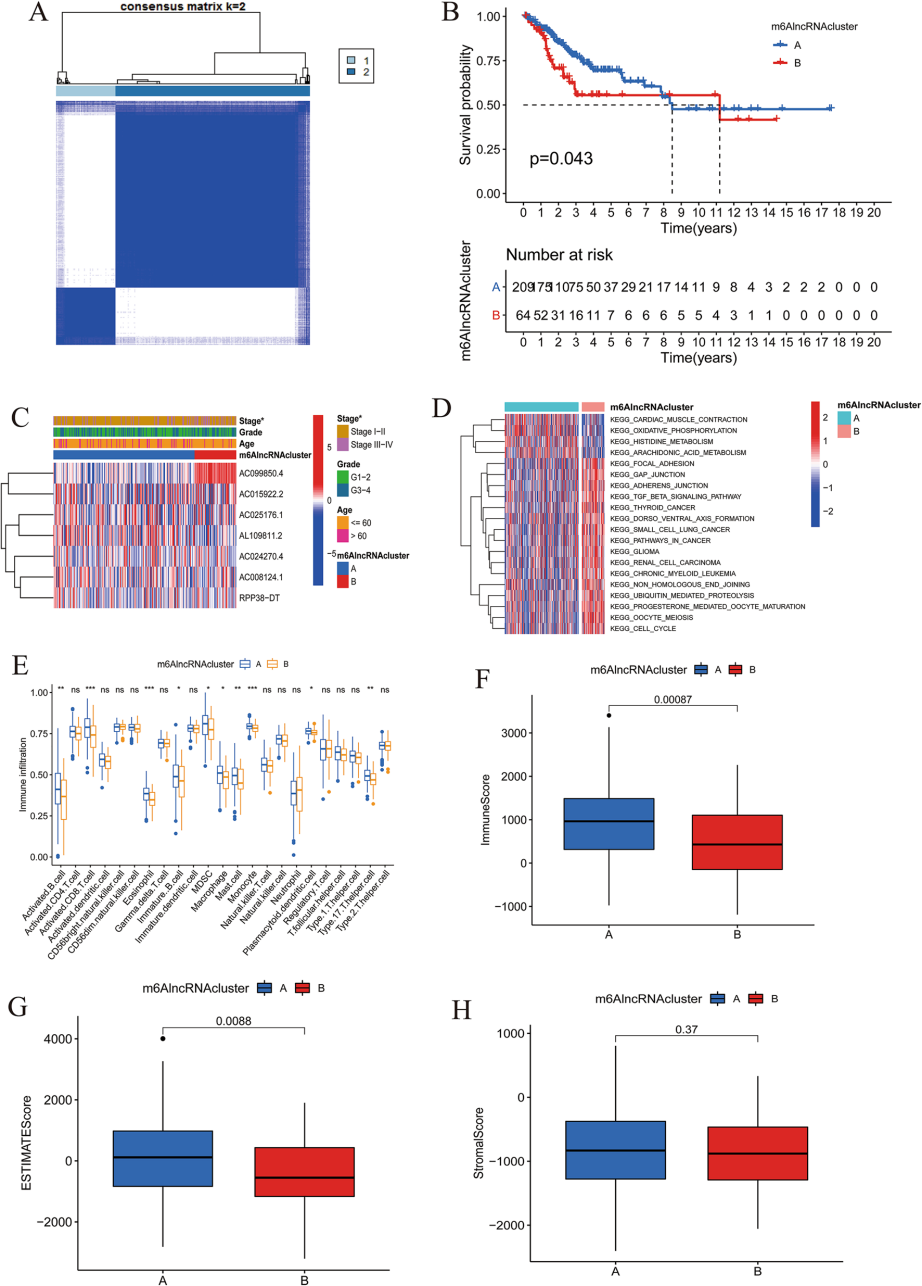
Differences in OS, clinicopathological and biological features, and TME characteristics between two different m^6^AlncRNA clustering patterns constructed based on 7 m^6^A-related prognostic lncRNAs. (**A**) Consensus clustering matrix for k = 2. (**B**) Kaplan–Meier curves of OS between m^6^AlncRNA clusterA and m^6^AlncRNA clusterB. (**C**) Heatmap and clinicopathological features of two m^6^AlncRNA clusters (*, *P* < 0.05). (**D**) Heatmap and the activation states of biological pathways in two different m^6^AlncRNA clustering patterns. (**E**) The abundance of each TME infiltrating cell in two m^6^AlncRNA clustering patterns (*, *P* < 0.05; **, *P* < 0.01; ***, *P* < 0.001). (**F**) Immune score, (**G**) ESTIMATE score, and (**H**) stromal score in two m^6^AlncRNA clusters.

The “ConsensusClusterPlus” package, using the 7 m^6^A-related prognostic lncRNAs, was utilized to explore the molecular subtypes of patients. According to the cumulative distribution function (CDF), the area under the CDF curve, the tracking plot from k = 2 to 9 (Supplementary Fig. 2a-c), and the number of cases in any cluster cannot be too small, the k = 2 was identified as the cluster number in our study to divide patients into two different m^6^A-related lncRNA clustering patterns (Fig. 1a), including 209 cases in m^6^AlncRNA clusterA and 64 cases in m^6^AlncRNA clusterB. m^6^AlncRNA clusterA had a notably better outcome compared with clusterB (Fig. 1b). In addition, the heatmap revealed that m^6^AlncRNA clusterB was preferentially related to a high FIGO stage (Fig. 1c).

### TME immune infiltration characteristics of different m^6^AlncRNA clustering patterns

GSVA was applied to explore the biological behaviors between different m^6^AlncRNA clustering patterns. m^6^AlncRNA clusterA presented enrichment pathways related to oxidative phosphorylation, cardiac muscle contraction, histidine catabolism, and arachidonic acid metabolism (Fig. 1d). m^6^AlncRNA clusterB was significantly enriched in immune evasion, stromal, and tumorigenic activation pathways such as TGF beta signaling pathway, ubiquitin mediated proteolysis, focal adhesion, and pathways in cancer. Subsequently, we further compared TME cell infiltrates between two m^6^AlncRNA clusters. ClusterA showed higher infiltration levels of multiple immune cells such as activated B cell and activated CD8 T cell than clusterB (Fig. 1e). The TME cell-infiltrating characteristic of clusterA was consist with its matching survival advantage. As expected, clusterA exhibited higher immune score (Fig. 1f) and ESTIMATE score (Fig. 1g), suggesting that clusterA had a significantly higher immune cell content and lower tumor purity. However, no significant difference of stromal score was displayed between two clusters (Fig. 1h). These results indicated that the two distinct m^6^AlncRNA clustering patterns had markedly different TME.

### Generation of m^6^AlncRNA genes and KEGG pathway enrichment analysis

**Figure 2:**
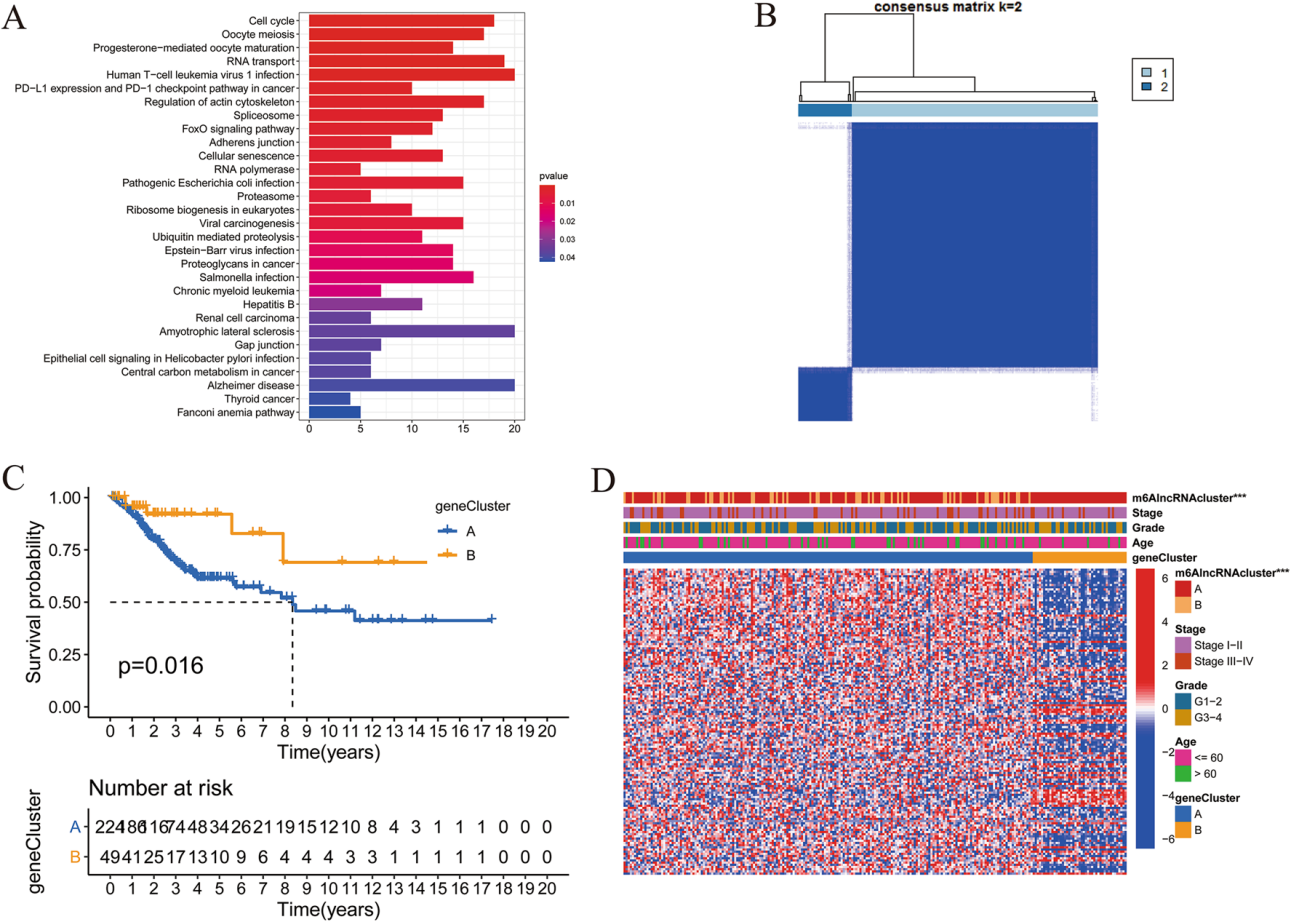
KEGG pathway analysis of DEGs between two m^6^AlncRNA clustering patterns and differences in OS and clinicopathological characteristics between two gene clusters constructed based on m^6^AlncRNA prognostic genes. (**A**) KEGG pathway analysis for DEGs between two m^6^AlncRNA clusters. (**B**) Consensus clustering matrix of m^6^AlncRNA prognostic genes for k = 2. (**C**) Kaplan–Meier curves of OS between gene clusterA and gene clusterB. (**D**) Heatmap and clinicopathological characteristics of two gene clusters (***, *P* < 0.001).

To further explore the potential biological behaviors of each m^6^AlncRNA clustering pattern, we determined 786 DEGs between two m^6^AlncRNA clusters using the limma package, named as m^6^AlncRNA genes. Then, we used the clusterProfiler package to perform KEGG enrichment analysis for the DEGs. Figure 2a showed the pathways with significant enrichment. To our surprise, these genes presented enrichment of pathways associated with PD-L1 expression and PD-1 checkpoint pathway and infection-related pathways such as viral carcinogenesis and Epstein-Barr virus infection. Afterward, we utilized univariate Cox regression analysis to explore the effect of DEGs on the survival of patients. Among the 786 genes, 140 were positively or negatively related to the OS with *P* value < 0.05, regarded as m^6^AlncRNA prognostic genes (Supplementary Table 3).

### Consensus clustering of m^6^AlncRNA prognostic genes

To further assess the regulation mechanism of m^6^AlncRNA clustering pattern in CC, we subsequently performed consensus clustering analysis based on the 140 prognostic DEGs so as to divide patients. The consensus clustering of the 140 m^6^AlncRNA prognostic genes classified patients into two different genomic subtypes, considered as gene clusterA (n = 224) and gene clusterB (n = 49), respectively (Supplementary Fig. 3a-c and Fig. 2b). We found that 4 out of the 7 m^6^A-related prognostic lncRNAs showed significantly different expression levels in the two gene clusters (Supplementary Fig. 4a). Gene clusterB had significantly better prognosis than gene clusterA (Fig. 2c). Moreover, the heatmap showed gene clusterA was preferentially associated with m^6^AlncRNA clusterB (Fig. 2d).

### Generation of m^6^AlncRNAscore and prognostic value

**Figure 3:**
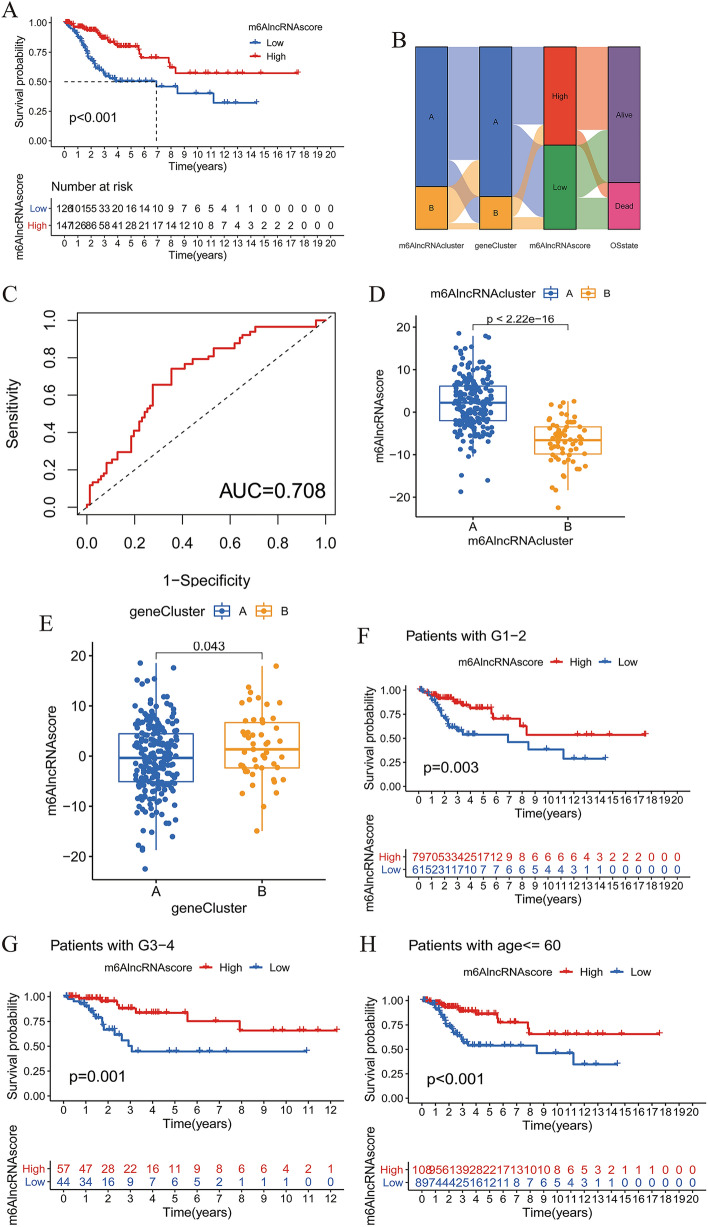

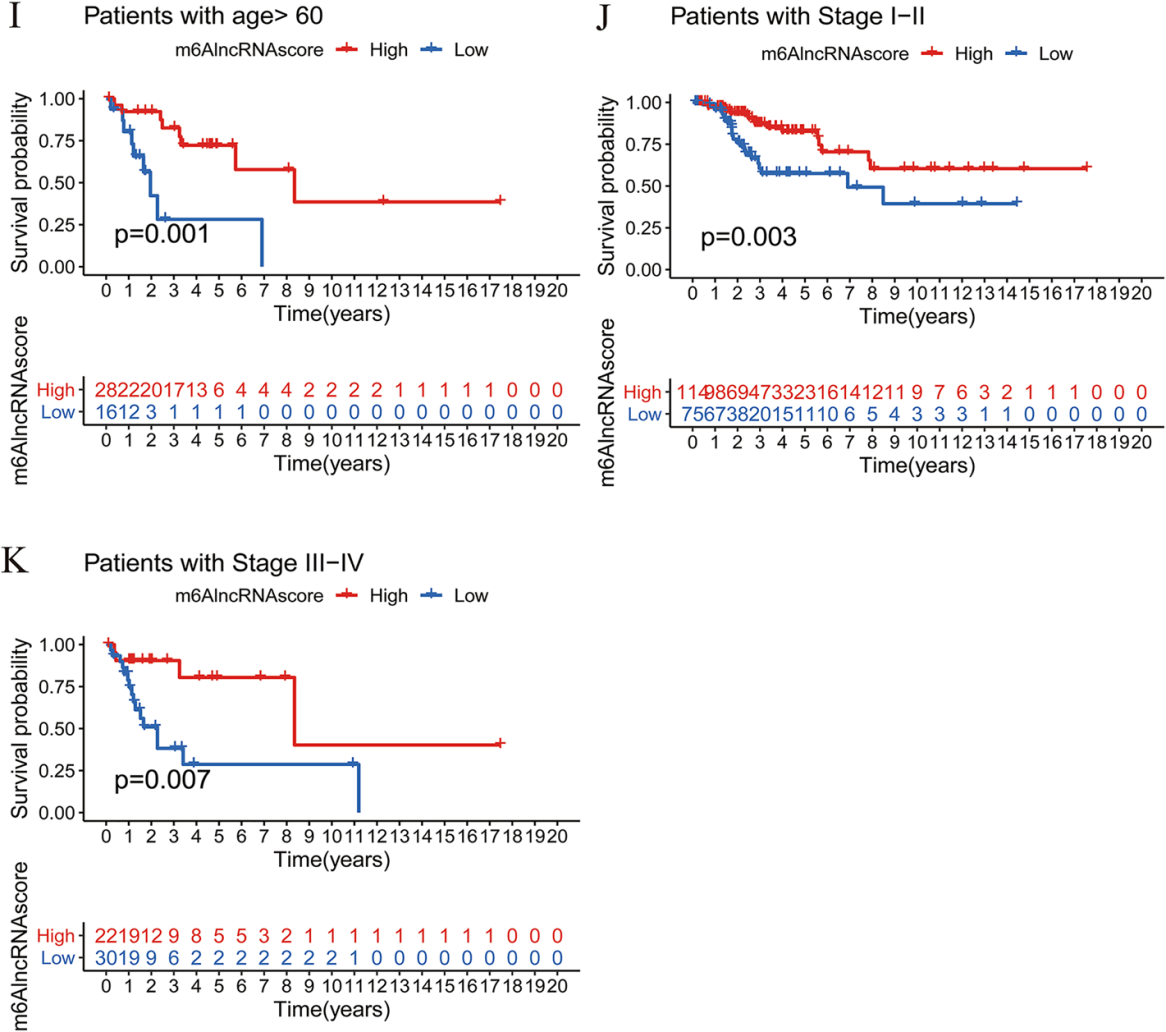
Construction of m^6^AlncRNAscore and prognostic value. (**A**) Kaplan–Meier curves of OS between the high- and low-m^6^AlncRNAscore groups. (**B**) Alluvial diagram showing the changes of m^6^AlncRNA clusters, gene clusters, m^6^AlncRNAscore, and survival state. (**C**) The 3-year ROC curve of m^6^AlncRNAscore in the OS. (**D**) Comparison of m^6^AlncRNAscore between two m^6^AlncRNA clusters. (**E**) Comparison of m^6^AlncRNAscore in two gene clusters. Kaplan–Meier curves of OS between the high- and low-m^6^AlncRNAscore groups in (**F**) grade1/2 patients, (**G**) grade3/4 patients, (**H**) patients with age ≤ 60 years, (**I**) patients with age > 60 years, (**J**) stage I/II patients, and (**K**) stage III/IV patients.

To reveal the role of 140 prognostic DEGs in CC, we used PCA to construct a scoring system to quantify the m^6^AlncRNA clustering pattern in each patient, termed as m^6^AlncRNAscore. We then divided patients into the high-m^6^AlncRNAscore group (n = 147) and the low-m^6^AlncRNAscore group (n = 126) according to the cutoff value -0.85 determined by the survminer package. Of the 7 m^6^A-related prognostic lncRNAs, 6 displayed significantly different levels between two different m^6^AlncRNAscore groups (Supplementary Fig. 4b). A better prognosis was observed in the high-m^6^AlncRNAscore subgroup (Fig. 3a). The alluvial diagram showed the corresponding relationship between m^6^AlncRNA cluster grouping, gene cluster grouping, m^6^AlncRNAscore grouping, and survival outcomes (Fig. 3b). The matching rates of m^6^AlncRNA clusterA with high-m^6^AlncRNAscore and m^6^AlncRNA clusterB with low-m^6^AlncRNAscore were 67.5% and 90.6%, respectively. To assess the accuracy of m^6^AlncRNAscore in predicting the OS, we performed ROC analysis and found that the 3-year AUC value was 0.708, implying that m^6^AlncRNAscore had a good prognostic discrimination performance (Fig. 3c). Subsequently, we compared the m^6^AlncRNAscore between different clustering subtypes. The m^6^AlncRNAscore in m^6^AlncRNA clusterA, as expected, was dramatically higher than that in m^6^AlncRNA clusterB (Fig. 3d). Similarly, gene cluster B had significantly higher m^6^AlncRNAscore compared with gene cluster A (Fig. 3e). Further stratified survival analysis results showed that the OS time in the low-m^6^AlncRNAscore group was dramatically shorter compared with the high-m^6^AlncRNAscore group, no matter for patients with grade1/2, grade3/4, age ≤ 60 years, age > 60 years, stage I/II, or stage III/IV (Fig. 3f-k).

### Independent prognostic value of m^6^AlncRNAscore in the prognosis of CC

**Figure 4:**
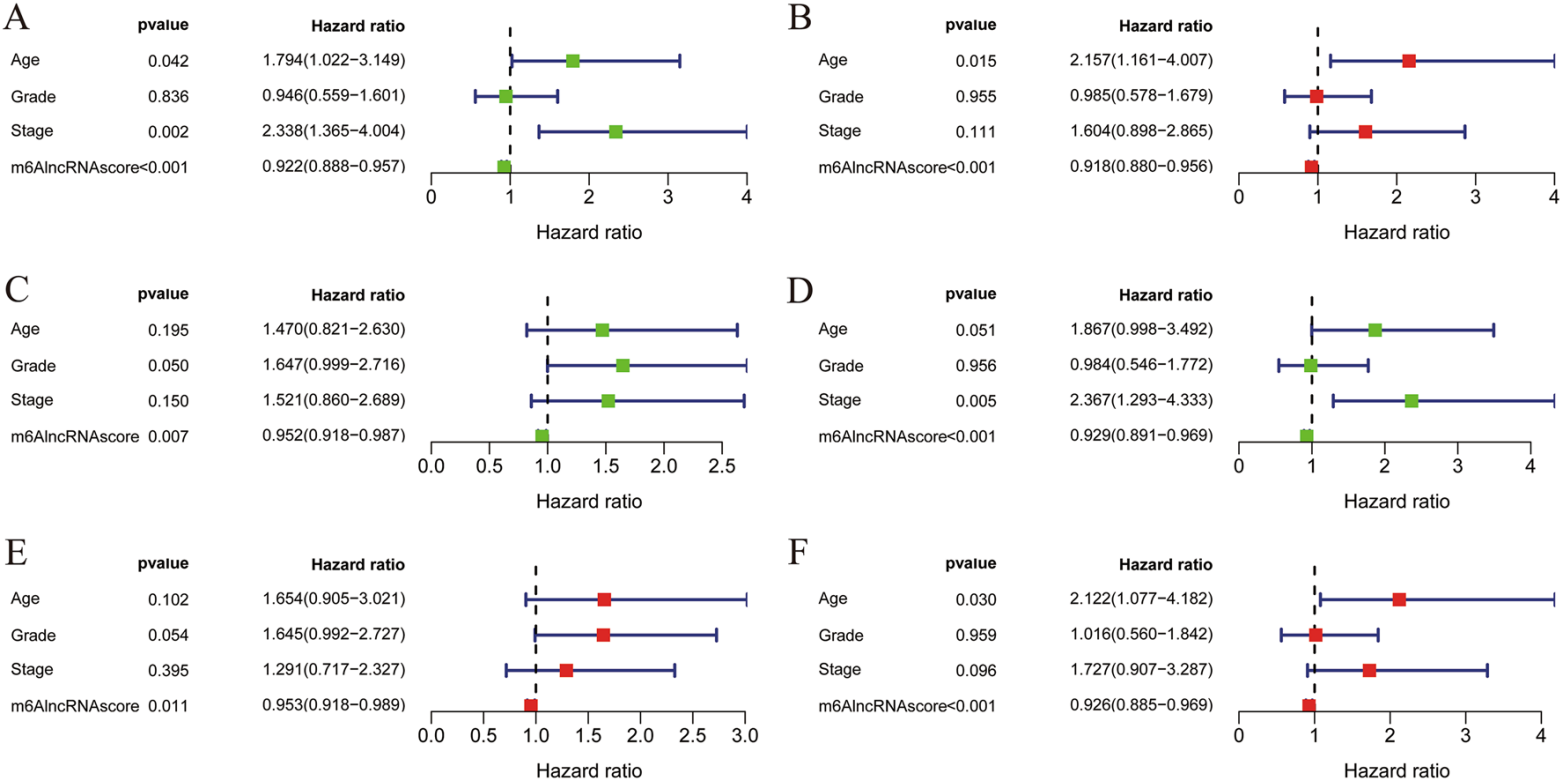
Independent prognostic value of m^6^AlncRNAscore in CC patients. (**A**) Univariate and (**B**) multivariate Cox regression analyses for the OS. Univariate Cox regression analyses for the (**C**) PFS and (**D**) DSS. Multivariate Cox regression analyses for the (**E**) PFS and (**F**) DSS.

As shown in Fig. 4a, the univariate Cox analysis results showed that m^6^AlncRNAscore, age, and FIGO stage were significantly related to the OS of CC patients. Subsequent multivariate Cox analysis results displayed that age was an independent risky factor (HR = 2.157, *P* value = 0.015), but m^6^AlncRNAscore was an independent protective factor (HR = 0.918, *P* value < 0.001) for the OS of CC patients (Fig. 4b).

Considering the significance of PFS and DSS in tumor prognosis, we further validated the prognostic value of m^6^AlncRNAscore in the PFS and DSS. In the univariate analysis, high-m^6^AlncRNAscore was significantly associated with better PFS (Fig. 4c) and DSS (Fig. 4d). Moreover, further multivariate Cox regression analysis results showed that m^6^AlncRNAscore was not only an independent prognostic factor for the PFS (Fig. 4e), but also an independent prognostic factor for the DSS (Fig. 4f). Our results strongly indicated that m^6^AlncRNAscore had good prognostic value in CC.

### TME immune infiltration characteristics of different m^6^AlncRNAscore groups

**Figure 5:**
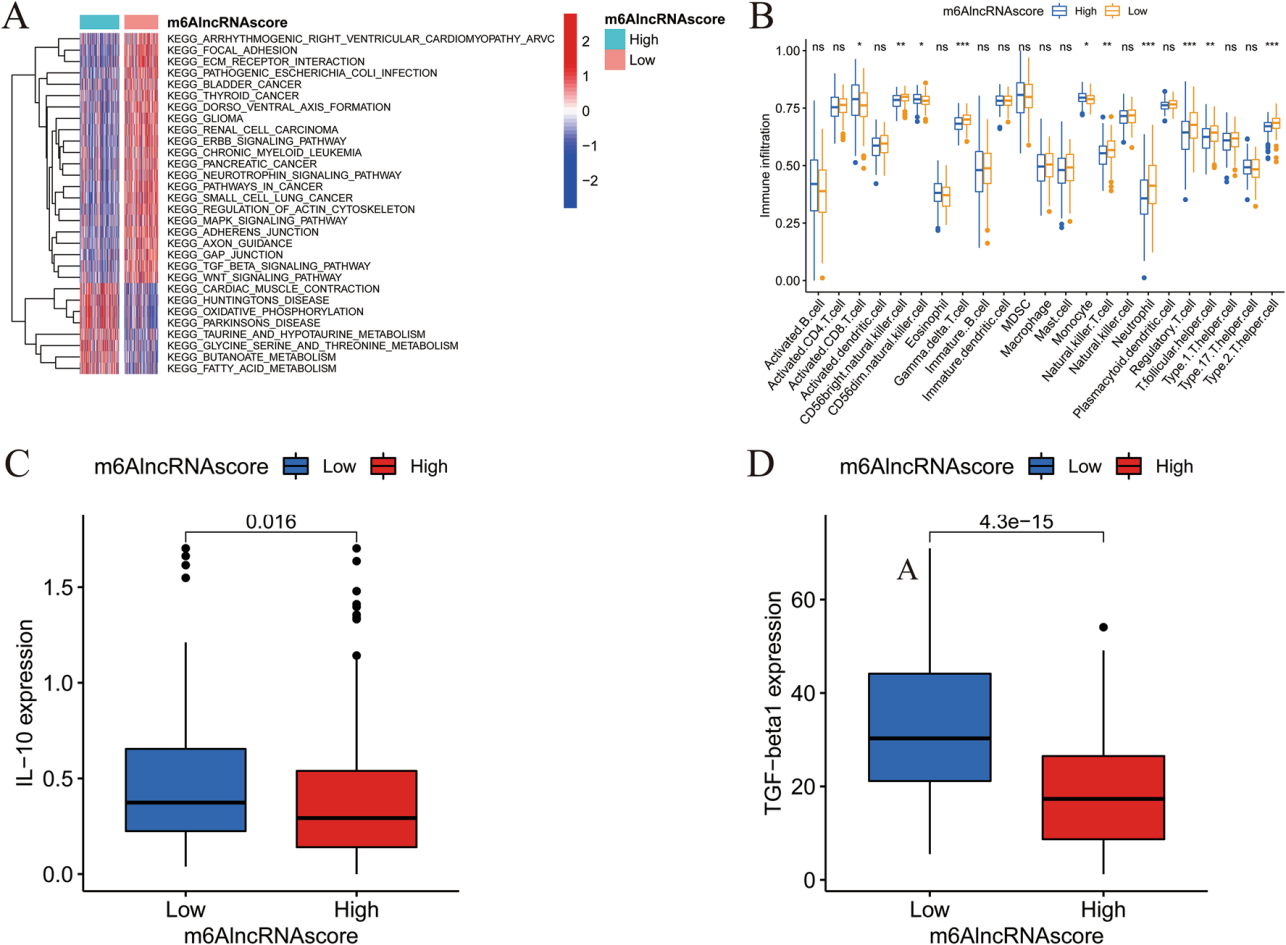
TME cell infiltration characteristics in the high- and low-m^6^AlncRNAscore groups. (**A**) Heatmap and the activation states of biological pathways in two m^6^AlncRNAscore groups. (**B**) The abundance of each TME infiltrating cell in two m^6^AlncRNAscore groups (*, *P* < 0.05; **, *P* < 0.01; ***, *P* < 0.001). The expression levels of (**C**) IL-10 and (**D**) TGF-beta1 in two m^6^AlncRNAscore groups.

To verify the biological behaviors of m^6^AlncRNA clustering patterns in TME, we performed GSVA and ssGSEA analyses in two m^6^AlncRNAscore groups. The high-m^6^AlncRNAscore group was characterized by enrichment of hallmark pathways such as oxidative phosphorylation and cardiac muscle contraction (Fig. 5a) and infiltration of activated CD8 T cell, CD56dim natural killer cell, and monocyte (Fig. 5b). The low-m^6^AlncRNAscore group was characterized by enrichment of immunosuppressive, stromal, and carcinogenic activation pathways such as wnt signaling pathway, TGF beta signaling pathway, MAPK signaling pathway, ERBB signaling pathway, focal adhesion, extracellular matrix (ECM)-receptor interaction, and pathways in cancer (Fig. 5a). Besides, the low-m^6^AlncRNAscore group was rich in T helper 2 (Th2) and regulatory T (Treg) cells, two types of tumor immunosuppressive T cells (Fig. 5b). We then explored the expression profiles of immunosuppressive factors IL-10 (Fig. 5c) and TGF-beta1 (Fig. 5d) and found that their levels in the low-m^6^AlncRNAscore group were significantly higher. The above results indicated again that m^6^A-related lncRNA clustering patterns played a vital role in shaping TME landscape.

### Clinical and somatic mutation characteristics of different m^6^AlncRNAscore groups

**Figure 6:**
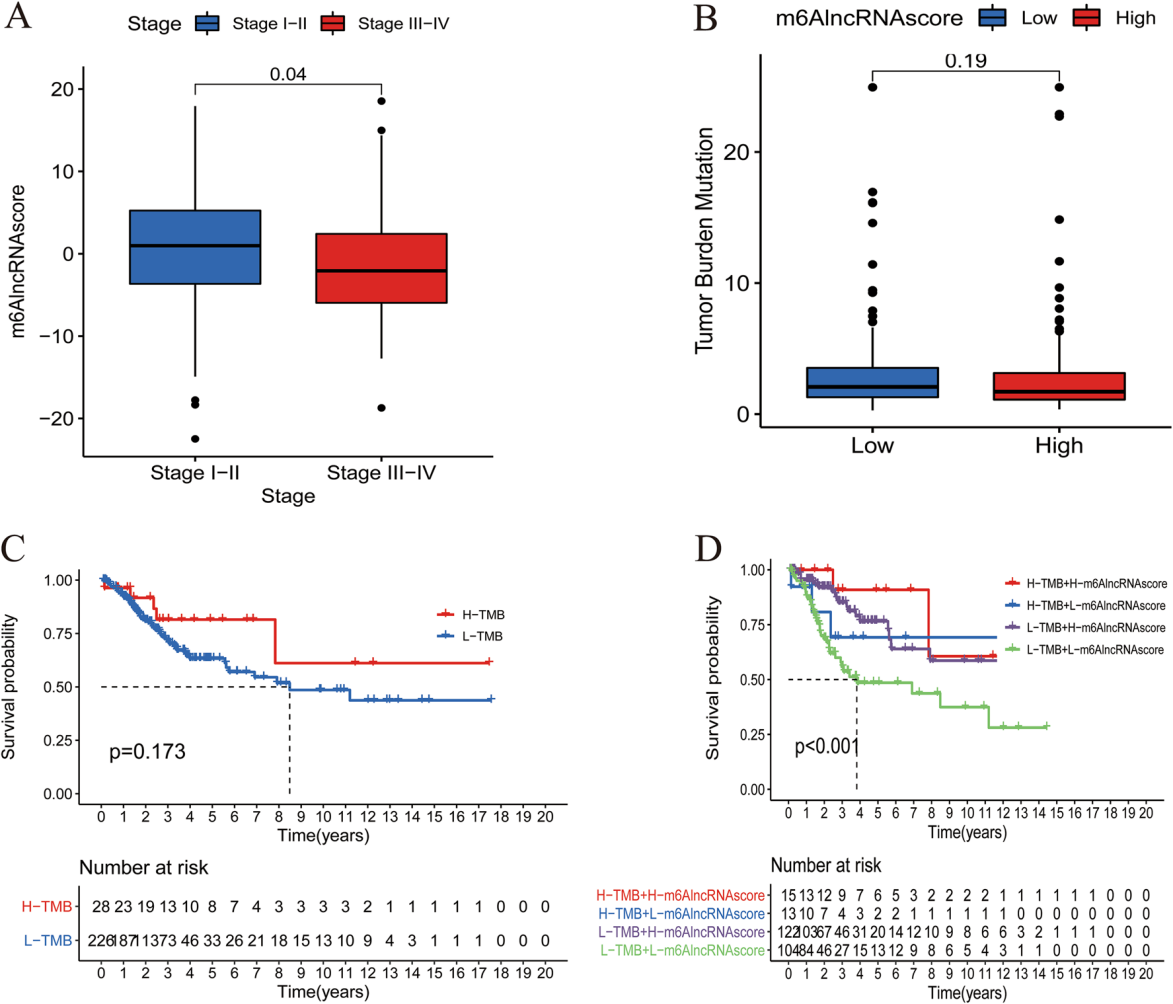
Clinical and somatic mutation characteristics in the high- and low-m^6^AlncRNAscore groups. (**A**) Comparison of m^6^AlncRNAscore between patients with stage I/II and patients with stage III/VI. (**B**) Comparison of TMB between the high- and low-m^6^AlncRNAscore groups. (**C**) Kaplan–Meier curves of OS in high- and low-TMB groups. (**D**) Survival analyses for subgroup patients stratified by m^6^AlncRNAscore and TMB using Kaplan–Meier curves.

As expected, the m^6^AlncRNAscore was significantly higher in patients with stage I/II than those with stage III/IV (Fig. 6a). However, no significant m^6^AlncRNAscore difference was observed in different age or grade subgroups. TMB quantification analysis results showed that the low-m^6^AlncRNAscore group presented no significant TBM difference in relative to the high-m^6^AlncRNAscore group (Fig. 6b). Next, the survminer package was applied to classify patients with information of somatic mutation and survival into the high-TMB group (n = 28) and the low-TMB group (n = 226) according to the cutoff value 6.32. A better prognostic tendency was observed in the high TMB group, while no significant difference was displayed between the high- and low-TMB groups (Fig. 6c). Moreover, we found that patients with low-m^6^AlncRNAscore and low-TMB had the worst prognosis, and the prediction power of m^6^AlncRNAscore was not disturbed by TMB during the first 5 years of follow-up (Fig. 6d).

### Patient’s response to ICIs in different m^6^AlncRNAscore groups

**Figure 7:**
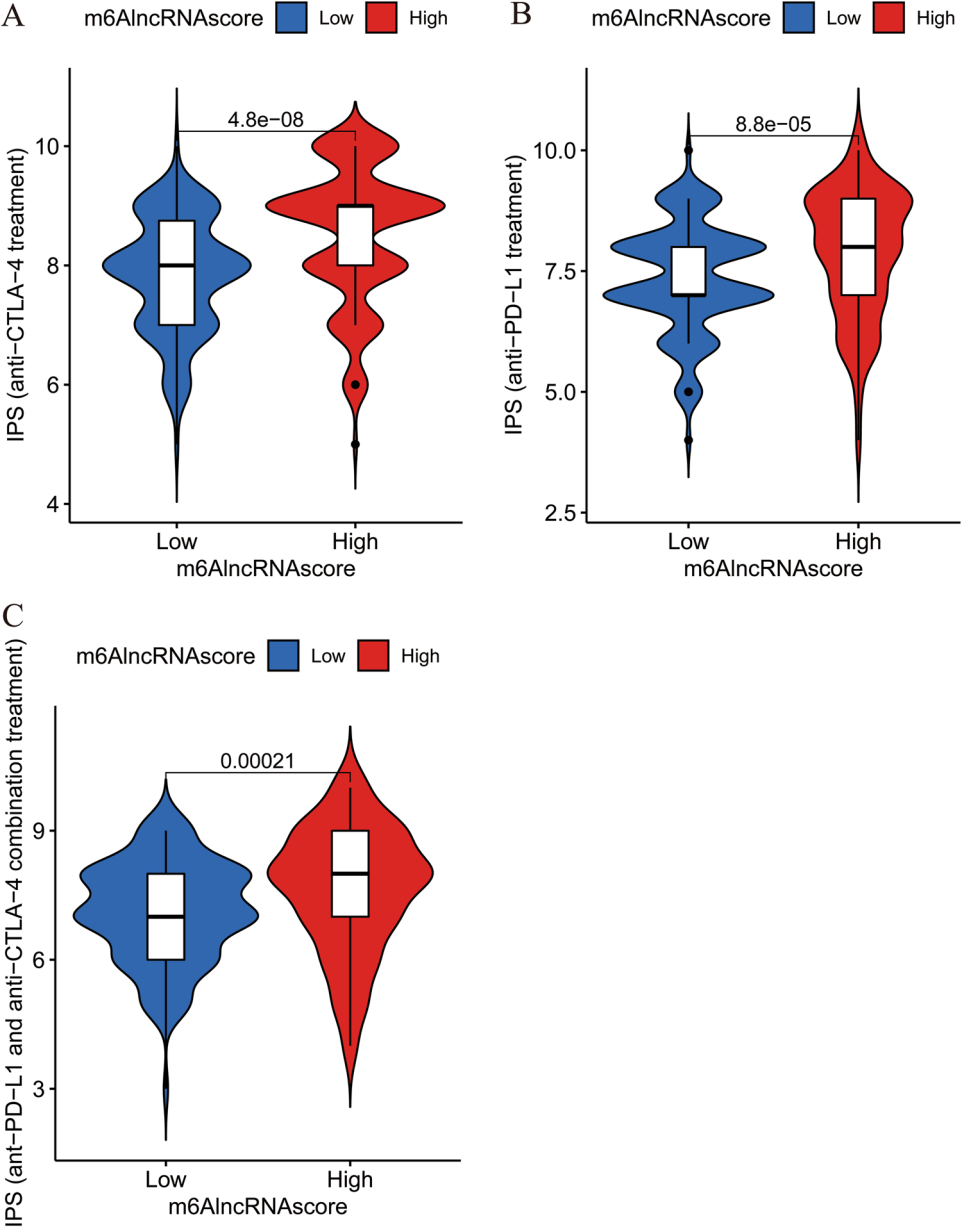
Comparison of IPS values between the high- and low-m^6^AlncRNAscore groups. Comparison of IPS values in (**A**) patients receiving anti-CTLA-4 treatment, (**B**) patients receiving anti-PD-L1 treatment, and (**C**) patients receiving anti-CTLA-4 and anti-PD-L1 combination treatment.

It has been reported that IPS values could predict the response of patients to ICIs. Whether patients received anti-CTLA-4 (Fig. 7a), anti-PD-L1 (Fig. 7b) or anti-CTLA-4 and anti-PD-L1 combination treatments (Fig. 7c), the IPS values of the high-m^6^AlncRNAscore group were dramatically higher compared with the low-m^6^AlncRNAscore group, suggesting that the corresponding ICI therapy responses in the high-m^6^AlncRNAscore group were significantly better than those of the low-m^6^AlncRNAscore group. These results indicated that patients with high-m^6^AlncRNAscore were more likely to benefit from ICIs.

### Drug sensitivity prediction in different m^6^AlncRNAscore groups

Then, we used the GDSC database to predict the valid drugs of high- and low-m^6^AlncRNAscore groups. Supplementary Fig. 5 showed that the CC patients in the high-m^6^AlncRNAscore group sensitively responded to 12 drugs (AZD3759, BI-2536, CDK95038, Dasatinib, ERK2440, Erlotinib, Gefitinib, Ibrutinib, NU7441, Osimertinib, Sapitinib, and UMI-77). The therapy responses to 18 drugs (Afuresertib, Axitinib, AZD6482, AZD8055, Dactolisib, GNE-317, GSK269962A, Ipatasertib, Leflunomide, MK-2206, Navitoclax, Nilotinib, OSI-027, Oxaliplatin, Palbociclib, PF-4708671, Ribociclib, and SB505124) in the low-m^6^AlncRNAscore group were significantly better than those of the high-m^6^AlncRNAscore group.

### Construction of the ceRNA network of the 7 m^6^A-related prognostic lncRNAs

To further identify the mechanism of the 7 m^6^A-related prognostic lncRNAs in CC patients, we constructed the lncRNAs-miRNAs-mRNAs ceRNA network. First, we obtained 30 miRNAs by the co-expression method. Then, miRanda, miRDB, miRTarBase and TargetScan software were used to identify 166 mRNA. Furthermore, we constructed and visualized the ceRNA network by incorporating 7 m^6^A-related prognostic lncRNAs, 30 miRNAs, and 166 mRNA (Supplementary Fig. 6).

### Validation of the expression levels of four m^6^A-related lncRNAs in CC samples

qRT-PCR assay was used to detect the expression of AC024270.4, AC008124.1, AC025176.1 and RPP38-DT in 6 tumor tissues and 6 normal samples. As shown in Supplementary Fig. 7, compared with normal tissues, cervical cancer tissues had higher AC024270.4 and AC008124.1, but lower AC025176.1. There was no difference in AC024270.4 expression between tumor samples and normal samples.

## Discussion

Increasing evidence indicates that TME plays an indispensable role in tumor immune escape and immunotherapy efficacy^5,6^. Therefore, identifying the role and potential regulatory mechanisms of m^6^A-related lncRNA clustering patterns in survival prediction and immune infiltration will deepen our understanding of tumor immune escape and enrich the effective population for cancer immunotherapy.

Here, we revealed two distinct m^6^AlncRNA clustering patterns based on 7 m^6^A-related prognostic lncRNAs. m^6^AlncRNA clusterA was characterized by low tumor purity and high infiltration level of immune cells, such as activated B cell and activated CD8 T cell, which are key effectors of anti-tumor immunity^24,25^. Further, m^6^AlncRNA clusterA were mainly involved in immune activation pathways such as oxidative phosphorylation, cardiac muscle contraction, and arachidonic acid metabolism. Inhibition of oxidative phosphorylation alone limits the proliferation of T cells exposed to persistent antigen and promotes T cell exhaustion by upregulating genes associated with T cell exhaustion^26^. Therefore, we speculated that the enriched oxidative phosphorylation signaling pathway in m^6^AlncRNA clusterA might promote the self-renewal of T cells, thereby enhancing antitumor immunity. Mediators released from arachidonic acid metabolic pathway play vital roles in maintaining the immune system normal function^27,28^. Cardiac muscle contraction pathway has been reported to be associated with autoimmune diseases characterized by abnormally activated immune response^29,30^. However, m^6^AlncRNA clusterB was characterized by enrichment of immune evasion and tumorigenic activation pathways such as TGF beta signaling pathway, ubiquitin mediated proteolysis, and pathways in cancer. Existing studies imply that TGF beta signaling inhibits not only the innate immunity but also the adaptive immune system, leading to tumor immune evasion and poor response to ICIs^31,32^. Thus, TGF beta signaling pathway is a potential tumor therapeutic target worthy of in-depth study^33^. Ubiquitin mediated proteolysis is involved in multiple biological processes including immune regulation and inflammatory response^34^. Melanoma patients with high level HECTD2, the E3 ubiquitin ligase involved in ubiquitin mediated proteolysis, had worse antitumor immunity and worse outcome of ICI treatment than those with low level HECTD2^34^. Consistent with TME immune infiltration characterizations, m^6^AlncRNA clusterA had better clinical outcomes in relative to m^6^AlncRNA clusterB, which was preferentially related to a higher FIGO stage.

Further, we explored the transcriptome difference between two m^6^AlncRNA clustering patterns. These DEGs were significantly linked to PD-L1 expression and PD-1 checkpoint pathway and immune-related infection pathways. PD-1/PD-L1 axis negatively regulates T cell activation by inhibiting Ras-Raf-MEK-ERK^35^. These findings demonstrated again that the m^6^AlncRNA clustering pattern dissimilarity was associated with tumor immunity difference. Moreover, we classified patients into two different genomic subtypes and two distinct m^6^AlncRNAscore groups based on the prognostic DEGs. Gene clusterA with poor prognosis was preferentially associated with m^6^AlncRNA clusterB with poor prognosis. To our surprise, up to 72.9% of patients had m^6^AlncRNA cluster grouping consistent with m^6^AlncRNAscore grouping. By integrated analyses, we found that the m^6^AlncRNAscore was a reliable and independent prognostic protective biomarker for the OS, PFS, and DSS of CC patients. No matter in the overall or in stratified survival analysis, patients with high-m6AlncRNAscore had better prognosis than low-m6AlncRNAscore. Previous studies have reported that high-TMB predicts a better clinical outcome and a higher ICI response rate in some tumors^36,37^. In our study, patients with high-TMB had longer OS than patients with low TMB, while no significant difference was observed. Although it could not be considered that the long-term prediction ability of m^6^AlncRNAscore was not affected by TMB due to the small sample size, we could determine that its prediction ability within 5 years was unaffected by TMB. Our findings indicated that m^6^AlncRNA clustering patterns might affect tumor immune escape by regulating TME, and finally affected the prognosis of patients.

Similar to GSVA results of m^6^AlncRNA clusters, the high-m^6^AlncRNAscore group was enriched in immune-inflamed pathways such as oxidative phosphorylation and cardiac muscle contraction, while the low-m^6^AlncRNAscore group was significantly related to immunosuppressive pathways such as wnt signaling pathway, TGF beta signaling pathway, and MAPK signaling pathway. As discussed earlier in this study, oxidative phosphorylation signaling and cardiac muscle contraction signaling are involved in activated immune response in humans^26,38,39^. TGF beta signaling^31–34,40^, Wnt signaling^41^, and MAPK signaling^42^ have been reported to promote tumor immune escape and limit antitumor immune response. Besides, the abundance of activated CD8 T cell and CD56dim natural killer cell was higher in the high-m^6^AlncRNAscore group. Consistent with the enrichment of immunosuppressive pathways, the fractions of Th2, Treg, IL-10, and TGF-beta1 were higher in patients with low-m^6^AlncRNAscore. Th2 and Treg are tumor immunosuppressive cells. Researchers have already found that patients with cervical cancer express higher Th2 and Treg than women with normal cervix^43^. Treg, a major barrier to effective anti-tumor immunotherapy, promotes tumor immune escape by production of immunosuppressiv cytokines such as IL-10 and TGF-beta^44,45^. TGF-beta in turn promotes the expansion of Treg^44,45^. It has been reported recently that m^6^A-related lncRNAs are novel prognostic biomarkers in lung cancer and breast cancer and are associated with TME^46,47^. Therefore, m^6^AlncRNAscore dissimilarity was significantly associated with TME difference. These findings could provide novel insights for cancer immunotherapy, that is, targeting m^6^A-related lncRNAs or m^6^A-related lncRNA relevant genes to reverse adverse TME, and then developing new immunotherapeutic drugs.

IPS comprehensively represents tumor immunogenicity and has been verified to predict the ICI treatment efficacy^19–23^. In our study, the high-m^6^AlncRNAscore group had higher IPS values than the low-m^6^AlncRNAscore group regardless of ICI therapy regimen, indicating that patients with high-m^6^AlncRNAscore might be more likely to benefit from ICIs. Additionally, IL-10 and TGF-beta might be potential new targets for anti-tumor immunotherapy in patients with high-m^6^AlncRNAscore, deserving further study. Furthermore, drug sensitivity analysis results found that the high-m^6^AlncRNAscore group had different sensitive drugs compared with the high-m^6^AlncRNAscore group. These findings demonstrated that m6AlncRNA clustering patterns could affect the therapeutic efficacy of ICIs.

Previous research on the 7 m^6^A-related lncRNAs was few. AC099850.4 was reported to be in relation to the prognosis of ovarian cancer through lncRNA-miRNA-mRNA competing triplets^48^. In our study, we also constructed the ceRNA network, which could help us better understand the mechanisms of the 7 m^6^A-related lncRNAs in CC. Consistent with the lower expression levels of AC024270.4 and AC008124.1 in cervical cancer tissues in relative to normal tissues in TCGA database, our qRT-PCR results displayed that AC024270.4 and AC008124.1 had significantly lower levels in tumor samples than normal tissues. The level of AC025176.1 in tumor samples was remarkably higher compared with normal tissues both in our experiment and TCGA analysis results. However, the expression of RPP38-DT in cervical cancer tissues was higher in TCGA database, but lower in our experiment in comparison with normal controls. The expression difference of RPP38-DT may be associated with the small sample size in TCGA-cervical cancer database with only three controls and our experiment with 6 pairs of case–control samples.

One limitation of our study was that our results were not validated in another database. Another limitation was that the full mechanisms of the 7 m^6^A-related lncRNAs in CC remained unclear. More research is needed in the future.

In summary, we firstly revealed the important regulation role of m^6^A-related lncRNA clustering patterns on prognosis, TME, and ICI therapeutic efficacy in CC. The difference of m^6^A-related lncRNA clustering patterns was a vital factor leading to the heterogeneity and complexity of individual TME. The systematic evaluation of m^6^A-related lncRNA clustering patterns will help improve our understanding of TME immune infiltration characteristics and might provide novel potential approaches for immunotherapy response prediction and patient prognostic stratification in CC.

## Supplementary Information

Supplementary Information 1.

Supplementary Information 2.

Supplementary Information 3.

Supplementary Information 4.

Supplementary Information 5.

Supplementary Information 6.

Supplementary Information 7.

Supplementary Information 8.

Supplementary Information 9.

## Data Availability

Publicly available datasets were analyzed in this study. This data can be found here: TCGA database (http://www.cancer.gov/tcga), TCIA database (https://tcia.at/)", and UCSC Xena database (https://xenabrowser.net/).
