# Supplementary figures and images for: Prediction of prognosis, immune infiltration and immunotherapy response with N6-methyladenosine-related lncRNA clustering patterns in cervical cancer
Source: Sci Rep. 2022 Oct 14;12:17256. doi: 10.1038/s41598-022-20162-2 (PMC9568557; doi:10.1038/s41598-022-20162-2)

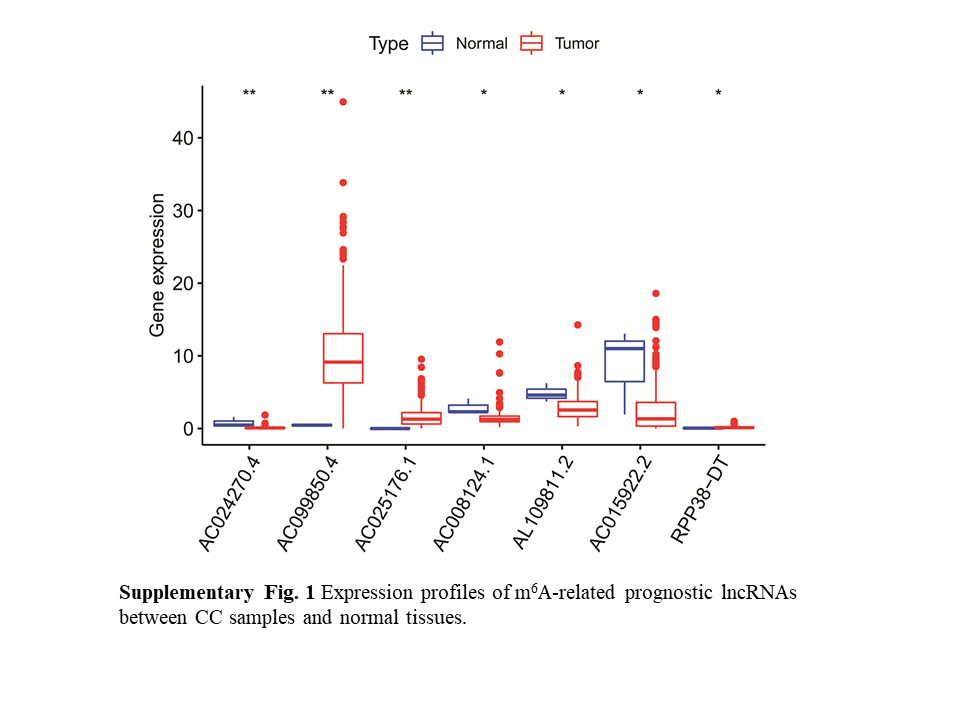

Supplement: Supplementary file 1 — Supplementary Information 1. [file 41598_2022_20162_MOESM1_ESM.tif]

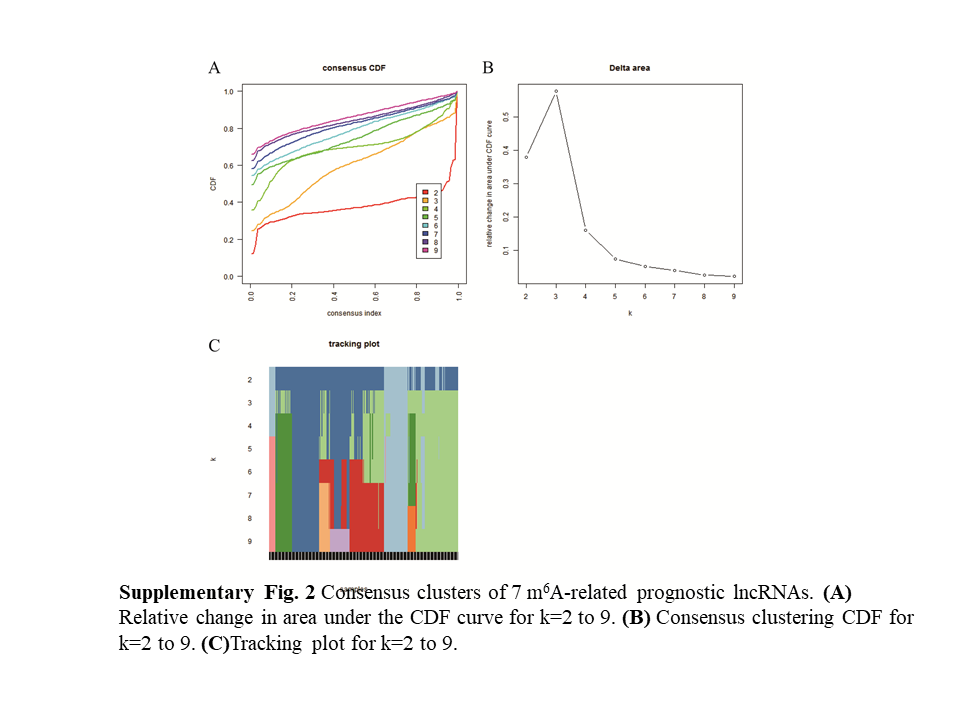

Supplement: Supplementary file 2 — Supplementary Information 2. [file 41598_2022_20162_MOESM2_ESM.tif]

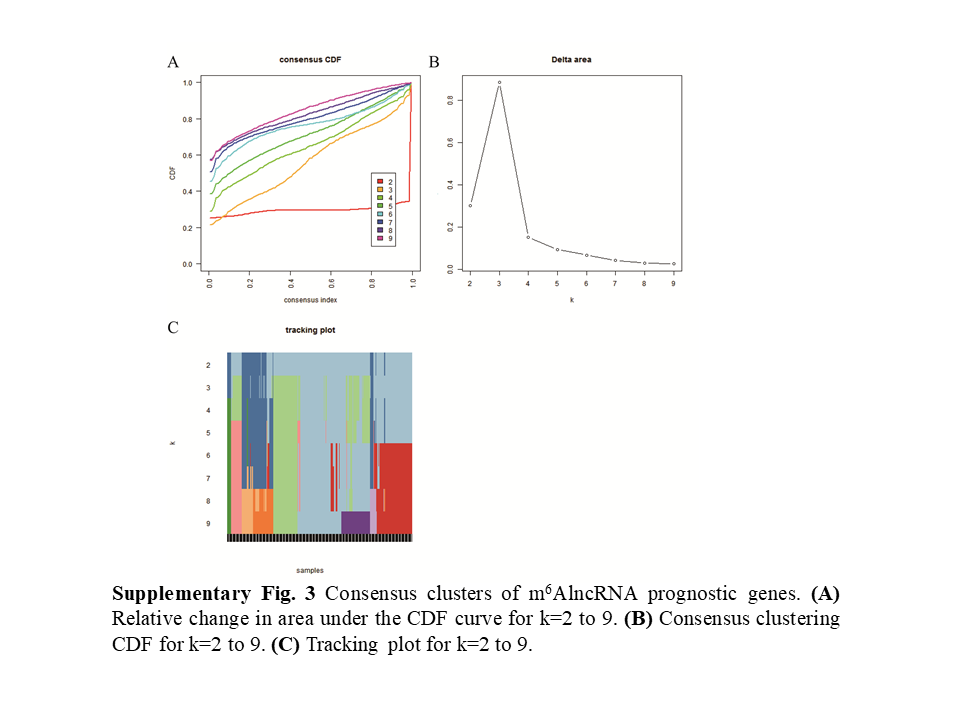

Supplement: Supplementary file 3 — Supplementary Information 3. [file 41598_2022_20162_MOESM3_ESM.tif]

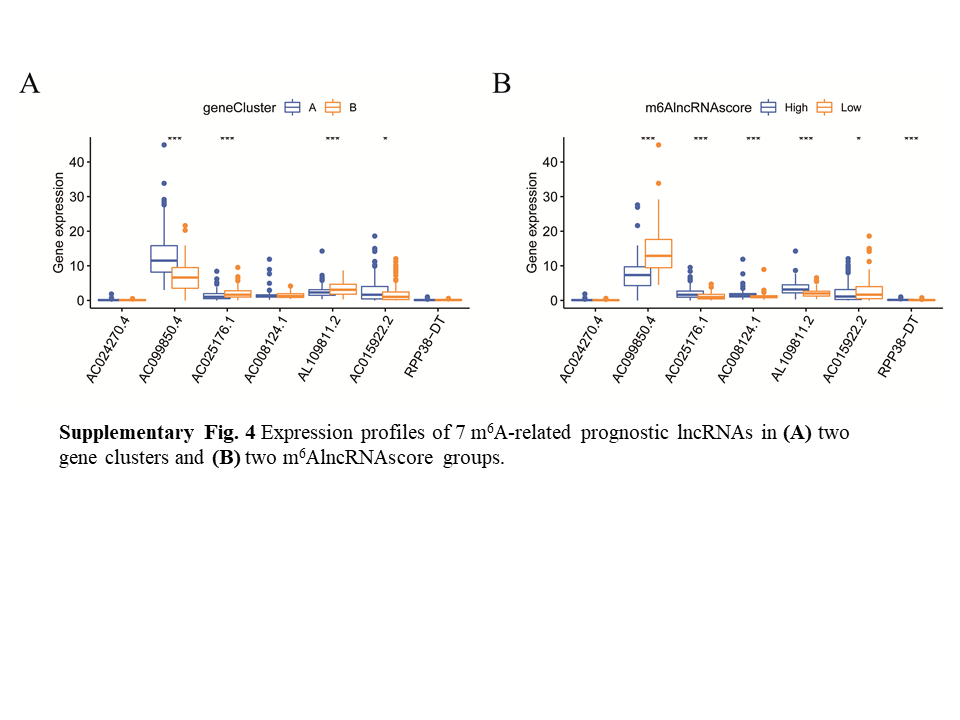

Supplement: Supplementary file 4 — Supplementary Information 4. [file 41598_2022_20162_MOESM4_ESM.tif]

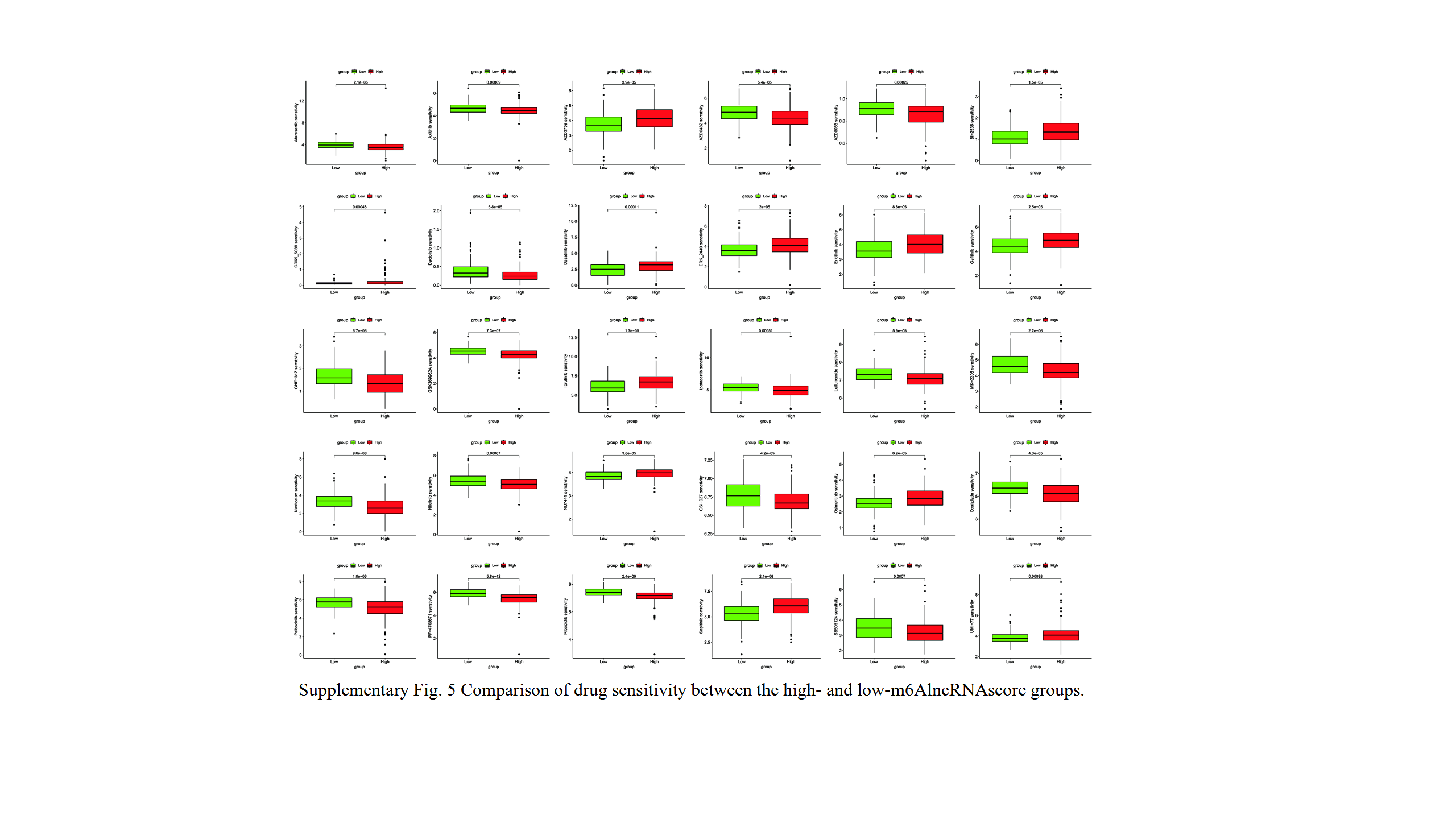

Supplement: Supplementary file 5 — Supplementary Information 5. [file 41598_2022_20162_MOESM5_ESM.tif]

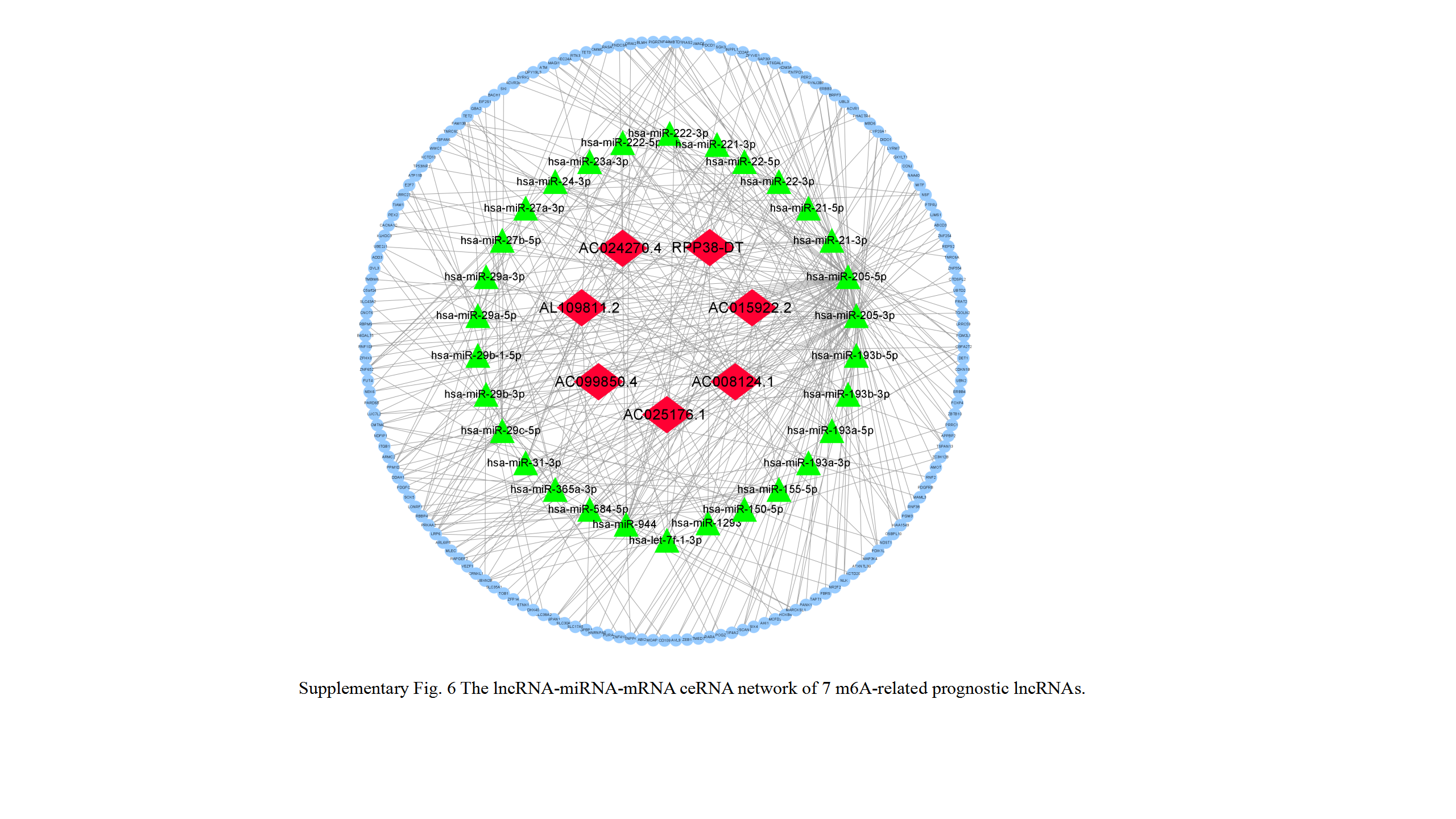

Supplement: Supplementary file 6 — Supplementary Information 6. [file 41598_2022_20162_MOESM6_ESM.tif]

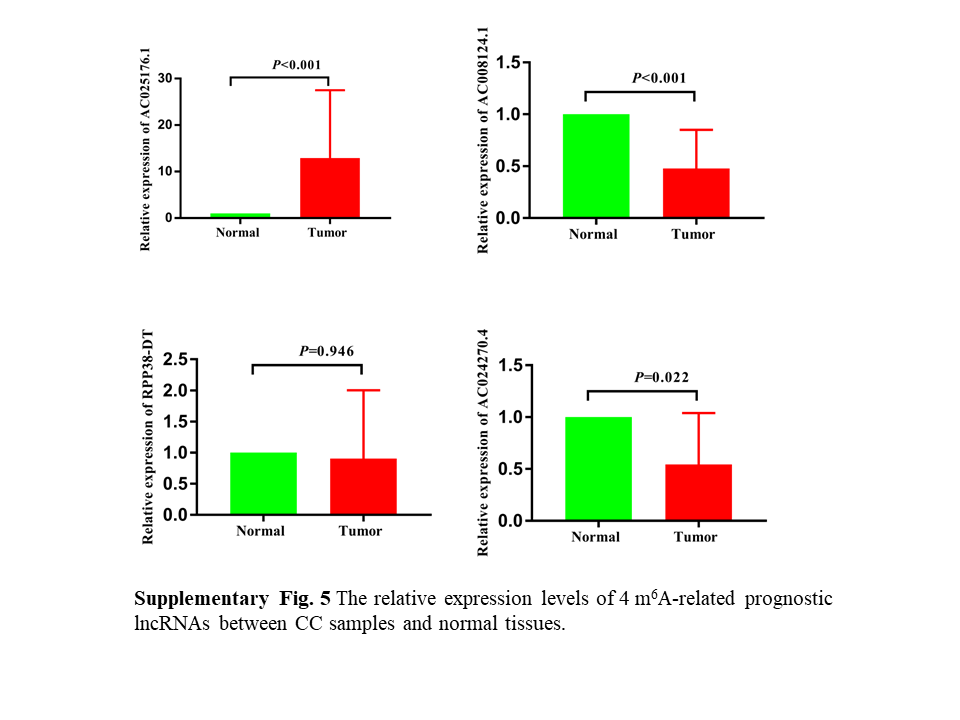

Supplement: Supplementary file 7 — Supplementary Information 7. [file 41598_2022_20162_MOESM7_ESM.tif]
