# Supplementary material for: Prediction of prognosis, immune infiltration and immunotherapy response with N6-methyladenosine-related lncRNA clustering patterns in cervical cancer
Source: Sci Rep. 2022 Oct 14;12:17256. doi: 10.1038/s41598-022-20162-2 (PMC9568557; doi:10.1038/s41598-022-20162-2)
Supplement: Supplementary file 8 — Supplementary Information 8. [file 41598_2022_20162_MOESM8_ESM.docx]

**Supplementary Table 1** The clinicopathological characteristics of CC patients

| Variables | No (%) |
| --- | --- |
| Age (years) |  |
| ≤ 60 | 246 (80.9) |
| > 60 | 58 (19.1) |
| Grade |  |
| G1 | 18 (5.9) |
| G2 | 136 (44.7) |
| G3 | 118 (38.8) |
| G4 | 1 (0.3) |
| NA | 31 (10.2) |
| FIGO stage |  |
| I | 162 (53.3) |
| II | 70 (23.0) |
| III | 45 (14.8) |
| IV | 21 (6.9) |
| NA | 6 (2.0) |
| Survival state |  |
| Alive | 234 (77.0) |
| Dead | 70 (23.0) |
| OS time (days) |  |
| ≤ 30 | 31 (10.2) |
| > 30 | 273 (89.8) |

NA, not available

Patients with OS less than or equal to 30 days were excluded in the survival analysis

**Supplementary Table 2** RT-PCR primers

| Gene name | Forward (5′ > 3′) | Reverse (5′ > 3′) |
| --- | --- | --- |
| RPP38-DT | CCATCGGAGTCGCTGCAAAGTC | AGGAGGAGGCTCATTAGGTCAGAAG |
| AC024270.4 | TCATGAGCCACGAAGTCAAGC | AGCCTTAAGTCTCAGGTCCTC |
| AC008124.1 | TGCCAACGACTTCTACCACCT | AGTCACCTCAGCTTTCCGTTC |
| AC025176.1 | CTTCAACTGGCTTCCTTGCTT | ACAGGAAACTCCTTCGTCACA |
