# Supplementary material for: Prediction of prognosis, immune infiltration and immunotherapy response with N6-methyladenosine-related lncRNA clustering patterns in cervical cancer
Source: Sci Rep. 2022 Oct 14;12:17256. doi: 10.1038/s41598-022-20162-2 (PMC9568557; doi:10.1038/s41598-022-20162-2)
Supplement: Supplementary file 9 — Supplementary Information 9. [file 41598_2022_20162_MOESM9_ESM.docx]

**Supplementary Table 3** The m^6^AlncRNA genes associated with the OS of CC patients

| m^6^AlncRNA genes | HR | HR.95L | HR.95H | *P* value |
| --- | --- | --- | --- | --- |
| AC099850.4 | 1.044217351 | 1.008225164 | 1.081494408 | 0.015619588 |
| ANO6 | 1.083816477 | 1.021445193 | 1.149996265 | 0.007776472 |
| ANLN | 1.032593133 | 1.010904934 | 1.054746637 | 0.003062553 |
| AK4P1 | 2.008025402 | 1.261141125 | 3.197236166 | 0.003307353 |
| FAM72B | 1.577795612 | 1.010725806 | 2.463021104 | 0.044758679 |
| MFAP3 | 1.231455736 | 1.046462353 | 1.449152208 | 0.012183507 |
| RECQL | 1.076775155 | 1.003291041 | 1.155641472 | 0.040260262 |
| FBXL18 | 1.366581666 | 1.124248703 | 1.661149748 | 0.001713234 |
| PTPN14 | 1.148128422 | 1.014448837 | 1.299423712 | 0.028735276 |
| B3GALNT2 | 1.188285534 | 1.03228592 | 1.367859895 | 0.016283906 |
| GART | 1.076851661 | 1.018063552 | 1.139034491 | 0.009738474 |
| POLR1B | 1.175649774 | 1.033303785 | 1.337605079 | 0.0139911 |
| HEATR1 | 1.10032632 | 1.019059267 | 1.188074188 | 0.014596218 |
| ITGB1 | 1.012922784 | 1.005144795 | 1.020760961 | 0.001095627 |
| PGAM1 | 1.032116743 | 1.011344874 | 1.053315243 | 0.002307571 |
| PMS2 | 1.357407685 | 1.088759373 | 1.692344212 | 0.00661316 |
| UBE2V1 | 1.429883623 | 1.059520611 | 1.929709675 | 0.019388625 |
| ASCC3 | 1.17144359 | 1.041834746 | 1.317176345 | 0.008168473 |
| AP000648.4 | 1.903653833 | 1.087618007 | 3.331958366 | 0.024193879 |
| YBX1P10 | 1.152473099 | 1.022747104 | 1.298653634 | 0.019852641 |
| AGO2 | 1.117077894 | 1.008100454 | 1.237835987 | 0.034514365 |
| NPEPPS | 1.073116634 | 1.011977553 | 1.137949461 | 0.018384667 |
| LGALS9 | 0.957477248 | 0.931186673 | 0.984510094 | 0.00222139 |
| FSCN1 | 1.0050904 | 1.002446449 | 1.007741325 | 0.000158011 |
| MYO1B | 1.02979598 | 1.01443315 | 1.045391469 | 0.000128898 |
| DSG2 | 1.014697231 | 1.006280975 | 1.023183878 | 0.000596109 |
| FASN | 1.008628026 | 1.001258719 | 1.016051572 | 0.021665674 |
| TNFRSF10D | 1.07561133 | 1.022916585 | 1.131020603 | 0.004454399 |
| NUS1P1 | 1.510509903 | 1.137201096 | 2.006364728 | 0.004404389 |
| NOCT | 1.143407323 | 1.029190917 | 1.27029911 | 0.012566411 |
| RAB3GAP2 | 1.212834557 | 1.041538678 | 1.412302484 | 0.012996733 |
| NAA60 | 0.845263461 | 0.737079474 | 0.969326027 | 0.016136187 |
| MIR4664 | 1.127280779 | 1.052207115 | 1.207710855 | 0.000656282 |
| FBXO11 | 1.17880347 | 1.028213751 | 1.351448199 | 0.018326698 |
| REEP3 | 1.048444112 | 1.009887557 | 1.08847272 | 0.013337 |
| DSE | 1.116944408 | 1.033524762 | 1.207097165 | 0.005228773 |
| PANX1 | 1.053974882 | 1.01877702 | 1.0903888 | 0.00241791 |
| GMFB | 1.10878703 | 1.005490698 | 1.222695228 | 0.038479544 |
| CEP170 | 1.341375788 | 1.077510364 | 1.669857724 | 0.008590104 |
| CHD7 | 1.274140512 | 1.069181625 | 1.518389398 | 0.00677836 |
| LDHAP4 | 1.157774536 | 1.048906952 | 1.277941646 | 0.003641485 |
| MAP4K4 | 1.047064931 | 1.002498901 | 1.093612141 | 0.038225529 |
| YPEL3 | 0.965723903 | 0.93535821 | 0.997075396 | 0.03238348 |
| G3BP1 | 1.057609676 | 1.002050186 | 1.116249707 | 0.041916194 |
| AKR1A1 | 0.976420458 | 0.963460636 | 0.989554607 | 0.000464889 |
| KLHL36 | 1.128054519 | 1.012197584 | 1.25717253 | 0.029314458 |
| DDN-AS1 | 2.74093258 | 1.220421433 | 6.155833715 | 0.014586099 |
| CNIH1 | 1.045244634 | 1.000579587 | 1.091903493 | 0.047036866 |
| SS18L2 | 0.951701755 | 0.912707444 | 0.992362049 | 0.020386185 |
| IARS2 | 1.02801949 | 1.00648131 | 1.050018575 | 0.010528336 |
| RGP1 | 1.108055972 | 1.017127994 | 1.20711262 | 0.018838601 |
| CCDC12 | 0.942579148 | 0.890266886 | 0.997965289 | 0.042368888 |
| PRRC2A | 1.017486052 | 1.001548378 | 1.033677343 | 0.031394349 |
| RASGEF1B | 0.668666776 | 0.510043817 | 0.876621267 | 0.003579041 |
| HOXA1 | 1.416242214 | 1.150895538 | 1.742766344 | 0.001010214 |
| ARHGAP8 | 2.429137416 | 1.345341299 | 4.386030957 | 0.00324065 |
| RNU1-72P | 1.97510674 | 1.019141657 | 3.827776647 | 0.043786462 |
| ERC1 | 1.117314524 | 1.004843612 | 1.242374167 | 0.040439991 |
| FUT11 | 1.166913509 | 1.069433251 | 1.273279222 | 0.000523923 |
| CHORDC1 | 1.197785807 | 1.03501244 | 1.386158063 | 0.015445951 |
| POM121 | 1.212434899 | 1.032508887 | 1.423714995 | 0.018758505 |
| PSMB10 | 0.980842143 | 0.965749684 | 0.996170462 | 0.014488371 |
| CYP51A1 | 1.30967055 | 1.013732995 | 1.692000712 | 0.03898602 |
| TGFB1 | 1.015513324 | 1.002853973 | 1.028332478 | 0.016161721 |
| PQBP1 | 0.976127089 | 0.957805661 | 0.99479898 | 0.012441794 |
| CIDECP1 | 0.681064765 | 0.504679386 | 0.91909681 | 0.012017847 |
| UBE3C | 1.057719333 | 1.007632683 | 1.110295653 | 0.023379826 |
| ABL2 | 1.290233733 | 1.084291395 | 1.535291245 | 0.004077841 |
| UXT | 0.985900296 | 0.973602054 | 0.998353886 | 0.026609384 |
| RBMS2P1 | 3.982158054 | 1.072547093 | 14.78497576 | 0.03896026 |
| ACTR2 | 1.014166577 | 1.00330872 | 1.025141939 | 0.010423835 |
| BCL9L | 1.083928806 | 1.01132329 | 1.161746861 | 0.022710875 |
| AL445305.1 | 3.477106195 | 1.196027684 | 10.10868532 | 0.02209572 |
| STX4 | 0.937950767 | 0.883828888 | 0.995386837 | 0.0346483 |
| AC092171.2 | 1.106606909 | 1.035165999 | 1.182978238 | 0.002929924 |
| AC063976.2 | 203.1599446 | 3.933589459 | 10492.69719 | 0.008278757 |
| ZNF281 | 1.144855544 | 1.044023183 | 1.255426353 | 0.004029849 |
| DIAPH3 | 1.18861222 | 1.047043287 | 1.349322447 | 0.007575146 |
| MTMR4 | 1.086322587 | 1.003187327 | 1.17634736 | 0.041519546 |
| RBAK | 1.164093261 | 1.080853673 | 1.253743363 | 5.97056E-05 |
| AL589880.1 | 1.36032543 | 1.068891471 | 1.731219048 | 0.012365166 |
| TSSK5P | 1.620553241 | 1.231081552 | 2.133240322 | 0.000576739 |
| CAD | 1.049606617 | 1.006104373 | 1.094989825 | 0.024977406 |
| ADCY1 | 1.966835776 | 1.204319101 | 3.212141172 | 0.00687515 |
| RNU5B-2P | 0.81246663 | 0.684448484 | 0.964429085 | 0.017597477 |
| P4HA1 | 1.019222664 | 1.010436324 | 1.028085407 | 1.63067E-05 |
| RELL1 | 1.13310803 | 1.010490861 | 1.270604076 | 0.03247108 |
| AC022382.1 | 0.074630194 | 0.015039678 | 0.370331445 | 0.001496321 |
| AC005840.4 | 0.761281898 | 0.597218403 | 0.970415723 | 0.027632747 |
| MAP2K1 | 1.043252438 | 1.008243947 | 1.079476502 | 0.015040524 |
| AIDA | 1.261144971 | 1.10583933 | 1.438261957 | 0.000539358 |
| NDUFA1 | 0.993895071 | 0.989277296 | 0.9985344 | 0.009959294 |
| PIEZO1 | 1.03659814 | 1.008849752 | 1.065109747 | 0.009420479 |
| HIGD2A | 0.993371557 | 0.987057025 | 0.999726487 | 0.040949584 |
| GDAP1 | 1.126748558 | 1.016275529 | 1.249230426 | 0.023414445 |
| FLT3LG | 0.596013154 | 0.415244705 | 0.85547552 | 0.005007758 |
| SLC4A3 | 1.171586554 | 1.044979743 | 1.31353269 | 0.006647453 |
| TIMM17B | 0.957544397 | 0.928549758 | 0.987444413 | 0.00568607 |
| AC024060.2 | 0.857846273 | 0.763335201 | 0.964059077 | 0.010036861 |
| SKI | 1.040853228 | 1.001391938 | 1.081869548 | 0.042305618 |
| COX7B | 0.984981416 | 0.972029146 | 0.998106275 | 0.025050148 |
| AC135507.1 | 0.247107453 | 0.074605705 | 0.818464134 | 0.022148554 |
| CENPS | 0.789940651 | 0.667913149 | 0.934262535 | 0.005883507 |
| RAB4B | 0.82114227 | 0.703910132 | 0.957898738 | 0.012168068 |
| TCP1 | 1.022601781 | 1.007240546 | 1.038197288 | 0.003801439 |
| EIF3C | 1.888461266 | 1.095214343 | 3.256244747 | 0.022186646 |
| EMC1 | 1.08543989 | 1.005513132 | 1.171719907 | 0.035654288 |
| SEPTIN7 | 1.082644585 | 1.01767928 | 1.151757062 | 0.011902518 |
| AC069282.1 | 1.152682702 | 1.008802513 | 1.317083765 | 0.036725955 |
| VANGL1 | 1.116204113 | 1.024926793 | 1.215610354 | 0.011549786 |
| NPM1P25 | 0.769463947 | 0.621867964 | 0.952090796 | 0.015874326 |
| MIRLET7F1 | 3.475158933 | 1.649983394 | 7.319303731 | 0.001046898 |
| AC115618.3 | 0.947650082 | 0.901609742 | 0.996041453 | 0.034339927 |
| GNA12 | 1.07184019 | 1.018656961 | 1.127800072 | 0.007543085 |
| HSPA8 | 1.002510665 | 1.000243409 | 1.00478306 | 0.029957872 |
| AC125807.2 | 1.340703941 | 1.050709299 | 1.710736793 | 0.018386397 |
| POLR3G | 1.177173988 | 1.044470753 | 1.326737579 | 0.007518563 |
| SSBP3 | 0.952162757 | 0.925309038 | 0.979795808 | 0.000784124 |
| FAM169A | 1.632148884 | 1.04544858 | 2.548102347 | 0.03112082 |
| SLC41A1 | 1.094546043 | 1.019326486 | 1.175316306 | 0.012885527 |
| AC113404.3 | 1.910845387 | 1.242094779 | 2.939654971 | 0.003214602 |
| SNORA71C | 1.253412978 | 1.05656835 | 1.486930868 | 0.009563114 |
| GANAB | 1.009090163 | 1.001870868 | 1.01636148 | 0.013504149 |
| AC073896.4 | 0.937493462 | 0.893607122 | 0.983535124 | 0.008323403 |
| ASAP2 | 1.093127867 | 1.012436827 | 1.180249968 | 0.022853199 |
| ADPRM | 0.752396298 | 0.607314631 | 0.932136589 | 0.00924251 |
| ZC3HAV1L | 1.168390553 | 1.0134991 | 1.34695382 | 0.031972396 |
| EHD2 | 1.011393437 | 1.002130591 | 1.0207419 | 0.015806767 |
| LYRM9 | 0.417234587 | 0.214453952 | 0.81175795 | 0.01004942 |
| RNF25 | 0.898839608 | 0.834978084 | 0.967585445 | 0.00456422 |
| OSMR | 1.022887754 | 1.006586 | 1.039453516 | 0.005765681 |
| PRSS53 | 1.77693078 | 1.10258221 | 2.863716619 | 0.018224436 |
| MSANTD3-TMEFF1 | 15202824.92 | 1971.538237 | 1.17231E+11 | 0.000293159 |
| AC107021.1 | 4.069344732 | 1.146641151 | 14.44180381 | 0.029878234 |
| SPTBN1 | 1.05382709 | 1.017001389 | 1.091986252 | 0.00386597 |
| AC026877.1 | 2.117830201 | 1.011201938 | 4.435518356 | 0.046646226 |
| SRPK2 | 1.098657874 | 1.007898097 | 1.197590438 | 0.03245231 |
| PSME2 | 0.9927953 | 0.985978183 | 0.999659552 | 0.039703325 |
